# Lessons Learned from COVID-19 Response in Correctional and Detention Facilities

**DOI:** 10.3201/eid3013.230776

**Published:** 2024-04

**Authors:** Caroline Waddell, Ashley Meehan, Megan Schoonveld, Zoe Kaplan, Michael Bien, Claire Bailey, Emily Mosites, Liesl M. Hagan

**Affiliations:** Centers for Disease Control and Prevention, Atlanta, Georgia, USA (C. Waddell, A. Meehan, M. Schoonveld, Z. Kaplan, C. Bailey, E. Mosites, L.M. Hagan);; CDC Foundation, Atlanta (M. Bien)

**Keywords:** COVID-19, SARS-CoV-2, coronavirus disease, severe acute respiratory syndrome coronavirus 2, viruses, respiratory infections, lessons learned, correctional health, jail, prison, public health

## Abstract

The COVID-19 pandemic disproportionately affected persons held in and working in correctional and detention facilities, causing facilities’ traditional priorities to shift when healthcare and public health needs temporarily drove many aspects of operations. During July–August 2022, we interviewed members of health departments and criminal justice organizations to document lessons learned from the COVID-19 response in correctional settings. Participants valued enhanced partnerships, flexibility, and innovation, as well as real-time data and corrections-specific public health guidance. Challenges included cross-sector collaborations, population density, scarcity of equipment and supplies, and mental health. Most participants reported improved relationships between criminal justice and public health organizations during the pandemic. Lessons from COVID-19 can be applied to everyday public health preparedness and emergency response in correctional facilities by ensuring representation of correctional health in public health strategy and practice and providing timely, data-driven, and partner-informed guidance tailored to correctional environments when public health needs arise.

During the COVID-19 pandemic, persons held in correctional and detention facilities in the United States experienced higher COVID-19 incidence and deaths than the general public (*1*,*2*). Dense housing conditions in those settings can increase the risk for rapid virus transmission among both persons held in and persons working in these facilities (*3*–*5*), and high prevalence of comorbidities among incarcerated persons can increase the risk for severe COVID-19 outcomes (*6*).

Because of elevated COVID-19 risk, public health agencies recommended enhanced prevention strategies for correctional and detention facilities (*7*). However, some strategies were difficult to implement or produced unintended consequences. For example, limiting in-person visitation and implementing quarantine and medical isolation in restrictive environments negatively affected mental health among incarcerated persons (*8*,*9*). In addition, such restrictive conditions discouraged persons from reporting COVID-19 symptoms, sometimes resulting in further transmission and large outbreaks (*10*).

During the pandemic, many correctional and detention facilities shifted operations to address healthcare and public health needs in addition to traditional security and public safety priorities. Over the extended period that these public health measures were in place, facilities had to find ways to balance COVID-19 prevention with ongoing security, mental health, and programmatic needs. To maintain this balance, facilities and public health agencies collaborated in unprecedented ways, sharing information and developing cross-disciplinary relationships (*11*).

Numerous editorial articles have highlighted the need to prioritize confinement facilities in future public health responses (*10*,*12*–*15*). In addition, published review articles emphasized the importance of collaborative approaches among public health and correctional agencies to address infectious diseases in correctional and detention facilities broadly (*16*,*17*). Several existing qualitative analyses included perspectives from primarily individual carceral systems, from multiple carceral systems at the same governmental level (i.e., state prisons), and from incarcerated persons regarding their unique needs during the COVID-19 response (*11*,*18*–*21*). However, empirical evidence is limited providing perspectives from a different types and levels of criminal justice organization types alongside viewpoints from healthcare organizations and health departments, all of whom play integral roles in infectious disease preparedness and response. In this analysis, we report findings from in-depth interviews with diverse public health and justice system organizations across the United States about the COVID-19 response in correctional settings. We identify and document common challenges, successful strategies, and actionable steps that public health practitioners can take to promote correctional health and to support correctional and detention facilities beyond COVID-19.

## Methods

### Participants

During July–August 2022, the Centers for Disease Control and Prevention (CDC) special populations team invited staff from criminal justice organizations and state health departments to participate in in-depth interviews related to their experiences responding to COVID-19 in correctional and detention facilities. The criminal justice organizations consisted of correctional and detention facilities, private correctional healthcare contractors, and federal agencies and professional organizations working within the US criminal justice system. Health department participants included staff assigned to respond to COVID-19 in correctional and detention facilities in their jurisdiction. We intentionally selected organizations (*22*) from an extensive list of governmental and nongovernmental organizations that the special populations team interacted with during the pandemic regarding COVID-19 response in correctional and detention facilities. To ensure that interviews included organizations without existing relationships with CDC, we supplemented the list with suggestions from leaders within prominent criminal justice organizations and agencies. The invited organizations were selected to maximize variation in geography, governmental level (federal, state, or local), facility size, population age, and role within the criminal justice system. Invited organizations could include <3 participants in the interview.

### Data Collection and Analysis

Two CDC staff (1 facilitator and 1 notetaker) conducted 1-hour virtual interviews. Interviewers stated that participation would not influence CDC funding or partnerships, and participants provided verbal informed consent. No incentives were provided. Interviewers used a semistructured questionnaire to explore challenges and successes during COVID-19 response in correctional and detention facilities, relationships between public health and criminal justice organizations, and ways public health agencies can support correctional health in the future.

Two reviewers analyzed the data using thematic analysis (*23*). Reviewers developed separate codebooks with a subset of interviews by using an inductive approach, compared findings, and grouped codes to identify broad themes (*24*). After coding all interviews, we synthesized responses into summaries of emergent themes. This activity was reviewed by CDC and was conducted consistent with applicable federal law and CDC policy (45 C.F.R. part 46.102(l) (*2*), 21 C.F.R. part 56; 42 U.S.C. §241(d); 5 U.S.C. §552a; 44 U.S.C. §3501 et seq).

## Results

Of the 33 invited organizations, 26 (79%) organizations (51 persons in total) participated in interviews. Participants included 21 criminal justice organizations and 5 state health departments (Table 1). Criminal justice staff roles included healthcare (46%), administration (29%), custody (22%), and occupational health (2%). All public health staff had served in an emergency response role related to correctional and detention facilities during the pandemic.

**Table 1:** Organization types and staff roles represented in COVID-19 lessons learned interviews, United States, July–August 2022

Category	No. (%)
Organization type	
State departments of health	5 (19%)
Criminal justice organizations	21 (81%)
Federal agencies within the U.S. Department of Justice	5 (24%)
State Departments of Corrections*	4 (19%)
Local jails†	4 (19%)
Youth detention and confinement facilities	3 (14%)
Professional organizations representing the criminal justice system	2 (10%)
Private healthcare contractors operating in correctional/detention facilities	2 (10%)
Private prison operators	1 (5%)
Total organizations interviewed	26 (100%)
Participant staff roles	
State health department participants	10 (20%)
Criminal justice organization participants	41 (80%)
Healthcare	19 (46%)
Administration	12 (29%)
Custody	9 (22%)
Occupational health	1 (2%)
Total staff included in interviews	51 (100%)

*Includes 1 unified system operating a state’s prisons and jails. †Participating jails included 1 small jail (<250 beds), 2 medium jails (250–1,000 beds), and 1 large jail (>1,000 beds), in addition to the jails represented in the 1 unified state system.

We describe lessons learned by presenting themes that emerged from participant interviews. The themes relate to participants’ views on facilitators and challenges to success in their COVID-19 response (Table 2; Figure; Appendix Table) and to opportunities for future collaboration between criminal justice and public health agencies.

**Table 2:** Illustrative quotes on select themes from COVID-19 lessons learned interviews with criminal justice organizations and state health departments, United States, July–August 2022*

Theme	Criminal justice participants	State health department participants
Operational innovations		
Operational innovations that facilities implemented in response to the pandemic, with value beyond COVID-19	“[COVID] Legitimized use of telehealth—before, payers didn't want to pay for it”	“Created library of addresses associated with correctional/detention facilities so we can match cases with addresses going forward… can reach out to facilities if cases pop up that haven’t been reported, to fill in gaps in reporting”
Leadership		
Role of leadership at multiple levels during pandemic response in correctional and detention facilities	“Clinical leadership is critical in these situations, and not often fostered in correctional settings.”	“Making sure there was someone in leadership meetings to advocate for resources for congregate settings—to make sure they didn’t get forgotten.”
Mental health		
Importance of mental health in public health emergency response; unintended consequences of COVID-19 prevention on the mental health of staff and people who were incarcerated	“Recognizing and appreciating staff - for wellness and burnout. Need to think about hazard pay, pay increases, recognizing the risks that staff face.”	“Investing in staff and making sure they are taken care of—wellness, time off, being flexible based on their needs, helping them feel supported and connected.”
Data capacity		
Having data systems in place for COVID-19 and beyond	“Ahead of the curve on mpox because COVID helped [us] prepare… Knowing we have these tools available and just have to make minor changes for a new disease makes [us] feel less stressed/overwhelmed when something new comes”	“One challenge [to pandemic response] is siloed data systems.”
Collaboration		
Internal and external partnerships with other criminal justice agencies, community-based organizations, court systems, and public health agencies	“When facilities were able to turn things around, it was about collaboration—not just across facilities, but within facilities, with health department, etc. multidisciplinary team to help figure out how to handle things.”	“Lots of opportunities to expand the relationships developed during COVID to other things. Working with the local jails now to become vaccination sites, training their nurses, getting grants to improve healthcare.”
Communication		
Internal and external communication, such as regular meetings, updates or education with colleagues and partners	“Close communication with local health department (don’t just call them when there’s an emergency) —keep maintaining that relationship, make sure you always have a contact”	“It's so important to take the time to have conversations to understand where facilities are coming from, why implementing public health recommendations was challenging, understanding why some recommendations are not feasible.”
Public health support		
Ways public health agencies can support correction and detention facilities in the future	“If public health understood life at a small city jail that would help. Everything seemed to flow well for the big jails, but small ones had it harder to make things work. Especially lack of on-site medical, no logistics section—these things have to be added to people’s existing duties.”	“The public health workforce needs to understand technical aspects of corrections—if scientists don’t know these things, that chips away at trust. We need technical training on what it is like to work in prison and jail.”

*See Appendix for an expanded version of this table.

**Figure:**
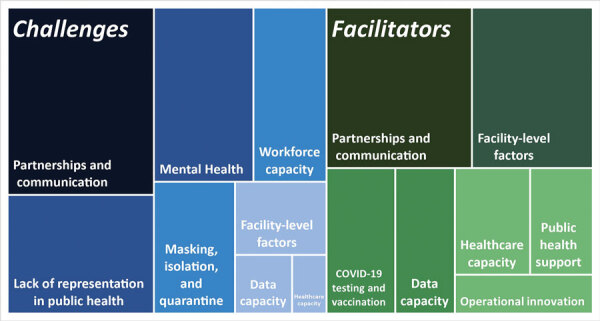
Facilitators and challenges to successful COVID-19 response in correctional and detention facilities, as reported by criminal justice organizations and state health departments, United States, July–August 2022.

### Facility-Level Factors and Operational Innovations

Participants reported that the population density inherent in correctional settings, limited isolation and quarantine space, and frequent movement of incarcerated persons between facilities and across jurisdictions complicated outbreak prevention and control. Given these constraints, participants stated that their response to COVID-19 was most successful when introducing operational innovation and flexibility into facility policies was possible and when leadership was strong and had previous experience in emergency planning.

The urgency of the pandemic enabled some criminal justice participants to develop innovative solutions to longstanding operational challenges and to gain support to continue them in the future. For example, some participants planned to expand medical screening at intake to include other infectious diseases or to continue using dedicated intake housing to improve uptake of rehabilitative programming and medical care at the beginning of confinement. Some participants (primarily from youth facilities and jails) planned to use diversion and decarceration strategies more intensively to avoid crowding and encourage community-based rehabilitation. Participants believed continuing to offer virtual services such as telehealth, virtual programming, and virtual visitation (originally implemented to maintain services amidst social distancing requirements) can increase access to rehabilitative programs and specialized healthcare going forward.

### Implementing COVID-19 Prevention Strategies in Correctional Settings

Criminal justice participants perceived testing and vaccination to be the most helpful COVID-19 prevention strategies, although they were difficult to implement. All facilities used testing to prevent introduction of COVID-19 into the population, and some used testing innovatively to maintain access to programming and education (test-to-program) during periods of the pandemic when testing supplies were sufficient. Although criminal justice participants believed masking and social distancing could prevent transmission, most felt implementation and enforcement were impractical in correctional environments, especially over long periods.

### Facility Healthcare Capacity

On-site healthcare capacity varied greatly across facilities. Larger facilities usually had sufficient healthcare services to manage most COVID-19 cases internally and conduct large-scale testing and vaccination programs. However, because those facilities are often not regarded as healthcare settings, their access to personal protective equipment and test kits was limited when supplies were constrained. For smaller facilities, especially jails, access to healthcare providers was limited or intermittent, increasing reliance on community hospitals and delaying testing and vaccination.

### Data Availability

Regardless of size and healthcare capacity, reliance on paper records was common and limited facilities’ ability to track population health, conduct contact tracing, access real-time data for decision-making, and comply with information requests for public health reporting, litigation, and government oversight. All health department participants expressed difficulty tracking COVID-19 cases and trends in correctional and detention facilities, particularly at the jail level, and none had systematic disease surveillance systems that included those facilities. Entering individual point-of-care test results was time-intensive for facility staff with competing responsibilities, particularly during mass testing events, limiting the data available to health departments. Most health departments relied on electronic laboratory reports, which required manual matching to addresses and provider names known to be associated with correctional and detention facilities.

### Workforce Capacity

Criminal justice participants reported that the pandemic exacerbated staffing shortages because of family care needs, fear of contracting COVID-19 in a congregate setting, and strict quarantine and isolation policies. Many facilities offered additional paid leave to encourage staff to stay home when sick and to allow for family care needs, but some expressed that abuse of those policies had been a challenge.

Health department participants reported that facilities’ COVID-19 consultation requests exceeded their capacity, contributing to staff burnout. Although their health departments allocated staff to corrections-specific roles, those positions were funded through time-limited sources, such as the COVID-19 American Rescue Plan Act and health equity grants, and participants expressed concern about funding sustainability for correctional health work within their health departments.

### Mental Health

All participants were concerned about mental health and low morale among incarcerated persons and staff because of COVID-related stress and trauma. In particular, participants mentioned that prolonged quarantine periods limited access to rehabilitative programming, visitation, and education for persons held in correctional and detention facilities, and they reported that this limited access sometimes led to increased suicide attempts and unrest. As mentioned, some facilities were eventually able to balance access to in-person services with disease prevention priorities through innovative testing approaches, where incarcerated persons exposed to COVID-19 were tested regularly and able to maintain in-person activities if they tested negative (test-to-program). Respondents stated that those approaches were only possible when they could consistently access rapid tests at low or no cost.

### Partnerships and Communication

All participants reported that partnerships and communication were imperative for success in their pandemic response. Examples included providing frequent updates to staff and incarcerated persons about policy changes, having regular meetings with external partners (e.g., community-based organizations, courts, public health agencies), and offering one-on-one education to maximize COVID-19 vaccine uptake. Participants emphasized unprecedented cross-disciplinary collaboration between facility healthcare and custody staff, building trust and respect between 2 missions that can sometimes be perceived as conflicting. Some participants expressed concern that those relationships could weaken once COVID-19 was no longer the common enemy.

Before the pandemic, no participating health departments had staff dedicated specifically to correctional health. At the time of the interviews, however, each had assigned from 0.25 to 2 full-time employees to address COVID-19 in correctional settings or to support correctional health broadly. All health department participants felt that their relationships with facilities improved during the pandemic, noting that having corrections-specific public health staff built trust that could enable disease prevention in the future. However, public health responses to COVID-19 cases were sometimes limited by the strength of relationships with individual facilities and by concerns that providing tailored guidance could involve health departments in litigation.

Although critical to success, collaboration and communication were challenging, particularly with external groups, such as the media, families of incarcerated persons, courts, and community hospitals concerned about absorbing facility case surges. The continual evolution of COVID-19 science and policy, combined with politicization of the pandemic, made managing misinformation difficult. Participants stated that in future public health emergencies, expectations should be set early that guidance will shift as understanding of the threat improves, especially for a novel disease like COVID-19.

### Participant Recommendations for Future Correctional Health Representation in Public Health

Although establishing productive collaborations and partnerships was one of the main challenges reported, most criminal justice participants also stated that their relationships with public health agencies improved during the pandemic, and some provided suggestions for actionable ways to expand and sustain them. Overall, participants believed that prioritizing and elevating correctional health within public health practice in future could reduce stigma that affects incarcerated persons and correctional staff. Specifically, criminal justice participants would like public health agencies at all levels of government to ensure that correctional health is represented within baseline strategy and operations, prioritize correctional and detention facilities when allocating resources, develop ways to track disease trends in correctional settings locally and nationally and share those data with the field, convene criminal justice partners to discuss shared health challenges, and disseminate information to the correctional health field early and consistently during public health emergencies. Participants believed that having a centralized point of contact and corrections-focused staff in public health agencies would support those needs.

In addition, participants cited corrections-specific public health guidance from CDC as a resource that supported their pandemic responses. However, many were frustrated that corrections-specific updates lagged behind guidance for other settings and that guidance was not written with front-line staff as an intended audience. Participants felt strongly that public health agencies should continue developing guidance and educational materials tailored for correctional settings and that such materials should include input from criminal justice partners and persons with lived experience of incarceration to improve their reach and relevance.

## Discussion

Interviews with criminal justice organizations and state health departments identified numerous lessons from the COVID-19 response in correctional and detention facilities, ranging from novel operational modifications to partnership strategies that expanded traditional ways of thinking within both sectors. Interviews with justice system organizations found that the pandemic response resulted in better communication and collaboration with nontraditional partners internally and externally, greater appreciation for public health data, and optimism about continued partnership with public health agencies. Participants reported that maintaining operational flexibility and openness to unique solutions enabled correctional and detention facilities of varying sizes and jurisdictional levels to overcome longstanding resistance to telehealth, virtual visitation, and population reduction. Those findings are consistent with a National Institute of Corrections report that included data from 31 state correctional agencies (*11*). Although innovative approaches such as regular testing to maintain access to programming (test-to-program) were available in some facilities represented here, a report by the Bureau of Justice Statistics found that most state correctional agencies suspended educational and visitation activities for extended periods of time during the pandemic, indicating the need for additional strategies to preserve access to those types of supports during future emergencies (*9*).

Key lessons for public health practitioners center on ensuring that correctional settings are better represented, prioritized for support during public health emergencies, and normalized as a major component of community health. Public health agencies can reach those goals by establishing and sustaining dedicated correctional health roles; those staff can work with criminal justice partners and persons who have been incarcerated to identify needs and codevelop corrections-specific public health tools for healthcare and nonhealthcare audiences. Greater public health awareness of correctional health should also lead to sustained investments in public health surveillance and data systems to include incarceration status and simplify facility case reporting. Public health participants voiced a need for more widely available funding for public health in correctional settings beyond time-limited emergency grants, noting that the success of future outbreak preparedness and response in those settings will depend on integrating correctional health into public health at all levels of government. Similar priorities for the future of infectious disease planning and response in correctional facilities have surfaced from other published literature as well; specifically, the need to include correctional health in everyday public health activities, including having staff and resources dedicated to these settings, developing tailored prevention strategies, and fostering proactive cross-sector collaborations (*11*,*12*,*18*).

The first limitation of our study is that, because CDC staff conducted the interviews, participants might have been hesitant to express critical views about CDC or other public health agencies. Second, staff members of many of the organizations interviewed had previously interacted with members of the research team in the context of COVID-19 emergency response, introducing selection bias. The views of persons from organizations without existing relationships with CDC might be underrepresented. Third, interviews did not include persons with lived experience of incarceration during the pandemic or their family members. Fourth, interview participants did not represent every type of correctional or detention facility in the United States. However, this work was not designed to be nationally representative, and our findings cover a wide range of perspectives across local, state, and federal government, representing a variety of roles in emergency response within correctional environments, as well as healthcare and professional organizations supporting those settings. We selected the sample carefully to ensure representatives from many types of correctional and detention settings, as well as health departments from across the United States, were included.

In conclusion, lessons from COVID-19 can improve everyday public health preparedness and emergency response in correctional settings. However, translating the pandemic-era elevation of public health priorities within correctional settings to a lasting cultural shift will depend largely on the ability of criminal justice and public health practitioners to maintain the bridges they built during the pandemic and on the public health system’s determination to dedicate sustained resources to correctional health.

Appendix Additional information about lessons learned from COVID-19 response in correctional and detention facilities.
