# Supplementary material for: Lessons Learned from COVID-19 Response in Correctional and Detention Facilities
Source: Emerg Infect Dis. 2024 Apr;30(Suppl 1):S5–S12. doi: 10.3201/eid3013.230776 (PMC10986833; doi:10.3201/eid3013.230776)
Supplement: Appendix — Additional information about lessons learned from COVID-19 response in correctional and detention facilities. [file 23-0776-Techapp-s1.pdf]

Article DOI: https://doi.org/10.3201/eid3013.230766

EID cannot ensure accessibility for supplementary materials supplied by authors. Readers who have difficulty accessing supplementary content should contact the authors for assistance.

Lessons Learned from COVID-19 Response in Correctional and Detention Facilities

Appendix

Appendix Table. Illustrative quotes on select themes from COVID-19 lessons learned interviews with criminal justice organizations and state health departments, United States, July–August 2022

Theme	Criminal Justice Participants	State Health Department Participants
Operational innovations<br>Operational innovations that facilities implemented in response to the pandemic, with value beyond COVID-19.	“[COVID] Legitimized use of telehealth - before, payers didn't want to pay for it”	“Created library of addresses associated with correctional/detention facilities so we can match cases with addresses going forward…can reach out to facilities if cases pop up that haven’t been reported, to fill in gaps in reporting”
	“Video visitation has expanded. So much more can be done virtually now. Court, visitation, telehealth.”<br>“Funding goes a long way to be able to buy equipment small jails otherwise can’t afford. Now there is more of a focus on cleaning overall, how well things are being cleaned.”	
Leadership<br>Role of leadership at multiple levels during pandemic response in correctional and detention facilities.	“Clinical leadership is critical in these situations, and not often fostered in correctional settings.”	“Making sure there was someone in leadership meetings to advocate for resources for congregate settings – to make sure they didn’t get forgotten.”
	“Empowered local facilities to adapt guidance to their needs.”	“Corrections was kind of on its own, didn’t have support from (public health) leadership”
Mental health<br>Importance of mental health in public health emergency response; unintended consequences of COVID-19 prevention on the mental health of staff and people who were incarcerated	“Recognizing and appreciating staff - for wellness and burnout. Need to think about hazard pay, pay increases, recognizing the risks that staff face.”	“Investing in staff and making sure they are taken care of - wellness, time off, being flexible based on their needs, helping them feel supported and connected.”
	“People with mental health concerns who didn’t have access to those services during COVID. (Jail is the) largest/only mental health provider in some places. Saw suicides go up, started a suicide prevention task force.”<br>“Everyone was doing the best they possibly could, but stresses continued to pile up. We modified services… but at the end of it people were still dying, and it felt like pushing a boulder uphill.”<br>“We are used to emergencies lasting 30, 60, 90 days… As soon as people start to breathe and transition and bring programs back,	“Trying to limit patient and staff deaths was an awesome responsibility, and understanding that many of the tools to do so are going to deprive them of their liberties.”

Theme	Criminal Justice Participants	State Health Department Participants
	another variant comes and deflates everyone."	
Data capacity<br>Having data systems in place for COVID-19 and beyond	"Ahead of the curve on mpox because COVID helped [us] prepare…Knowing we have these tools available and just have to make minor changes for a new disease makes [us] feel less stressed/overwhelmed when something new comes"	"One challenge [to pandemic response] is siloed data systems."
	"We gave local providers data and outcomes associated with their facilities. It helped get facilities to realize they can't justify not following infection control recommendations because of space or staff shortages."	"The DOC does not have an EHR. Health information exchange would be beneficial for corrections, but there could be a barrier for facilities to enroll if there is no benefit to them."
Collaboration<br>Internal and external partnerships with other criminal justice agencies, community-based organizations, court systems, and public health agencies	"When facilities were able to turn things around, it was about collaboration – not just across facilities, but within facilities, with health department, etc. multidisciplinary team to help figure out how to handle things."	"Lots of opportunities to expand the relationships developed during COVID to other things. Working with the local jails now to become vaccination sites, training their nurses, getting grants to improve healthcare."
	"Excellent collaboration between healthcare and security. We were mindful of each other's co-existing missions…with open communications about potential conflicts. Now we have a better understanding on how to work together in the future."	"Before the pandemic, our relationship with jails was cordial but distant. Working with the non-profit association for counties in our state helped us build relationships with county jails like never before. We've been able to establish ongoing communication, learn about each other and each other's roles, and gain access that hasn't existed before. When local public health did not blame facilities for things not going well, partnerships were more successful."
	"We brought in public health from every county and started collaborating – this has never happened before. The common enemy was COVID, and people just wanted to save lives."	"We offered facilities the opportunity to work with us to see where there were gaps that could be improved, but no one wanted to be on record because of liability. It's hard to give feedback for improvement in this kind of climate/culture."
	"Called upon skillful, creative people to think outside the box when their back was against the wall."	
Communication<br>Internal and external communication, such as regular meetings, updates or education with colleagues and partners	"Close communication with local health department (don't just call them when there's an emergency) – keep maintaining that relationship, make sure you always have a contact"	"It's so important to take the time to have conversations to understand where facilities are coming from, why implementing public health recommendations was challenging, understanding why some recommendations are not feasible."
	"We were trying to make the right decisions for the benefit of all, and having that be questioned is so hard. Now looking back, we didn't know then what we know now. The media is questioning those early decisions with today's information."	"Being able to provide facilities with guidance that is written for them feels inclusive and helps reinforce the message of information sharing bi-directionally. Having dedicated resources for corrections helps facilities feel like it is *theirs*."
	"In a population that is scared and doesn't know what is coming next, changing information can cause anger and fear and distrust."	
Public health support<br>Ways public health agencies can support correction and detention facilities in the future	"If public health understood life at a small city jail that would help. Everything seemed to flow well for the big jails, but small ones had it harder to make things work. Especially lack of on-site medical, no logistics section – these things have to be added to people's existing duties."	"The public health workforce needs to understand technical aspects of corrections – if scientists don't know these things, that chips away at trust. We need technical training on what it is like to work in prison and jail."
	"Have people with corrections experience advising on the impact of public health recommendations."	"It's important to follow through with corrections partners – we heard what you need and we're going to try to find a way to follow through on it. Come back with solutions, or let them know there aren't any"
